# Clinical applications of indocyanine green (ICG) enhanced fluorescence in laparoscopic surgery

**DOI:** 10.1007/s00464-014-3895-x

**Published:** 2014-10-11

**Authors:** Luigi Boni, Giulia David, Alberto Mangano, Gianlorenzo Dionigi, Stefano Rausei, Sebastiano Spampatti, Elisa Cassinotti, Abe Fingerhut

**Affiliations:** 1 Minimally Invasive Surgery Research Center, Department of Surgical and Morphological Sciences, University of Insubria, Varese, Italy; 2 Section for Surgical Research, Department of Surgery, Medical University of Graz, Graz, Austria; 3 First Department of Surgery, University of Athens, Hippokration University Hospital, Athens, Greece

**Keywords:** Laparoscopic surgery, Indocyanine green (ICG)-enhanced fluorescence, Near-infrared light (NIR), Cholecystectomy, Colorectal resection

## Abstract

**Background:**

Recently major developments in video imaging have been achieved: among these, the use of high definition and 3D imaging systems, and more recently indocyanine green (ICG) fluorescence imaging are emerging as major contributions to intraoperative decision making during surgical procedures. The aim of this study was to present our experience with different laparoscopic procedures using ICG fluorescence imaging.

**Patients and methods:**

108 ICG-enhanced fluorescence-guided laparoscopic procedures were performed: 52 laparoscopic cholecystectomies, 38 colorectal resections, 8 living-donor nephrectomies, 1 laparoscopic kidney autotransplantation, 3 inguino-iliac/obturator lymph node dissections for melanoma, and 6 miscellanea procedures. Visualization of structures was provided by a high definition stereoscopic camera connected to a 30° 10 mm scope equipped with a specific lens and light source emitting both visible and near infra-red (NIR) light (KARL STORZ GmbH & Co. KG, Tuttlingen, Germany). After injection of ICG, the system projected high-resolution NIR real-time images of blood flow in vessels and organs as well as highlighted biliary excretion .

**Results:**

No intraoperataive or injection-related adverse effects were reported, and the biliary/vascular anatomy was always clearly identified. The imaging system provided invaluable information to conduct a safe cholecystectomy and ensure adequate vascular supply for colectomy, nephrectomy, or find lymph nodes. There were no bile duct injuries or anastomotic leaks.

**Conclusions:**

In our experience, the ICG fluorescence imaging system seems to be simple, safe, and useful. The technique may well become a standard in the near future in view of its different diagnostic and oncological capabilities. Larger studies and more specific evaluations are needed to confirm its role and to address its disadvantages.

Major developments in minimal surgery video imaging have been achieved during the last few years: the use of high definition (HD) as well as 3-dimensional (3-D) systems has proved to be able to improve surgeon performance and, as consequence, patient safety [1–4].

Recently, indocyanine green (ICG)-enhanced fluorescence was introduced in laparoscopic surgery to improve the view and provide detailed anatomical information during surgery [5, 6].

ICG has been used in medicine since the late 50s [7–9] to measure cardiac output [10, 11], to study the anatomy of the retinal vessels [7], and to measure liver functional reserve before hepatic resection in cirrhotic livers [12].

The dye, ICG, can be injected into the human blood stream with practically no adverse effects [13]. ICG becomes fluorescent once excited with specific wavelength light in the near infra-red (NIR) spectrum (approximately 820 nm) [14] or a laser beam [15, 16]. The fluorescence can be detected using specific scopes and cameras and then transmitted to a standard monitor allowing identification of anatomical structures where the dye is present (i.e., biliary ducts, vessels, lymph nodes, etc.).

In this article, we present our experience in different laparoscopic procedures using ICG-enhanced fluorescence.

## Patients and methods

From January 2013 until May 2014, 108 ICG-enhanced fluorescence-guided laparoscopic procedures were performed at the Minimally Invasive Surgery Research Center of the Department of Surgical and Morphological Sciences of the University of Insubria (Varese, Italy).

These included 52 laparoscopic cholecystectomies, both for symptomatic lithiasis or acute cholecystitis, 38 colorectal resections both for benign and malignant diseases, 8 living-donor nephrectomies, three inguino-iliac/obturator lymph nodes dissection for lower limb melanoma, one laparoscopic kidney autotransplantation for renal artery transplantation, and six miscellaneous procedures (see below).

All the procedures were performed using indocyanine green (ICG-Pulsion^®^, Pulsion Medical Systems, Munich, Germany), diluted either with saline solution or albumin according to the procedure. Once the solution was prepared in the operating room, it was injected into a peripheral vein or around the tumoral area at a specific concentration according to the patient’s weight and clinical situation (see below).

### Indocyanine green

Indocyanine green is a sterile, anionic, water-soluble but relatively hydrophobic, tricarbocyanine molecule with a molecular mass of 776 Daltons.

ICG dye was developed for near infra-red (NIR) photography by the Kodak research laboratories in 1955 and was approved for clinical use in 1959 by the FDA [13].

Following intravenous injection, ICG is rapidly bound to plasma proteins, especially lipoproteins, with minimal leakage into the interstitium. There are no known metabolites. ICG is rapidly extracted by the liver without modifications and nearly exclusively excreted by the liver appearing unconjugated in the bile about 8 min after injection, depending on liver vascularization and function [13, 17].

When injected outside blood vessels, ICG binds to proteins and is found in the lymph, reaching the nearest draining lymph node usually within 15 min. After 1–2 h, it binds to the regional lymph nodes, deposited into macrophages [18–20].

The usual dose for standard clinical use (0.1–0.5 mg/ml/kg) [13] is well below the toxicity level.

ICG becomes fluorescent once excited either using a laser beam [15, 16] or by near infra-red (NIR) light at about 820 nm and longer wave lengths [14], the absorption peak is around 807 nm, and the emission peak is around 822 nm [13]. The fluorescence released by ICG can be detected using specifically designated scopes and camera.

### Laparoscopic equipment

In all cases, a laparoscopic system (KARL STORZ GmbH & Co. KG, Tuttlingen, Germany) was used. The imaging is generated by the high-end full high definition camera system (IMAGE 1 SPIES™, KARL STORZ) connected to a laparoscope with 30° field of direction and 10 mm diameter equipped with a specific filter for optimal detection of the NIR fluorescence and white light without manual switching. The powerful xenon light source (D-LIGHT P SCB, KARL STORZ) provides both visible and NIR excitation light. Switching from standard light to NIR is controlled by the surgeon by means of a pedal.

Visualization both in standard and NIR light is improved by a system of professional image enhancement (IMAGE 1 SPIES™ system, KARL STORZ GmbH & Co. KG, Tuttlingen, Germany) which offers adjustable visualization modalities that can be selected according to surgeon’s preferences.

### Timing and ICG dosage

Details of the timing and ICG doses used, varying slightly according to each procedure, are described below.

## Results

### Fluorescence-guided cholecystectomy

As ICG, once injected, concentrates in bile, it is possible to outline the biliary tree anatomy, especially in Calot’s triangle, by visualization under NIR light, during laparoscopic cholecystectomy, in both elective and acute settings.

The ICG dye was injected Intra-venously at least 15 min before surgery to allow ICG to concentrate in the bile [21, 22]. In our experience, the mean time between injection of ICG and surgery was 14 ± 9 min using a dose of 0.4 mg/ml/kg.

Laparoscopic cholecystectomy was performed in 52 patients (31 female and 21 male, mean age 53 ± 15 years), 35 for acute cholecystitis, and 17 for symptomatic cholelithiasis, all cases with four trocars using a standard technique [23].

**Fig. 1:**
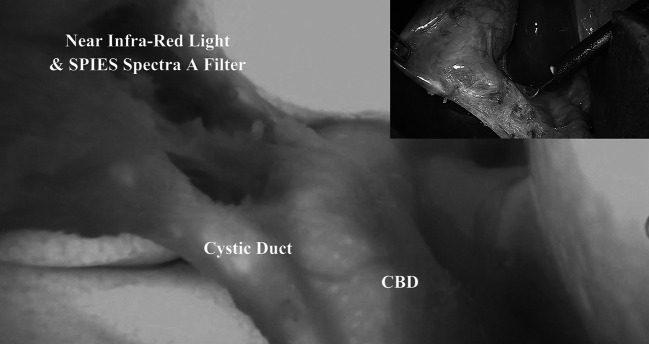
Identification of the biliary anatomy during laparoscopic cholecystectomy in non-acute setting. Insert in the *upper right corner* shows the operative view using a standard light

**Fig. 2:**
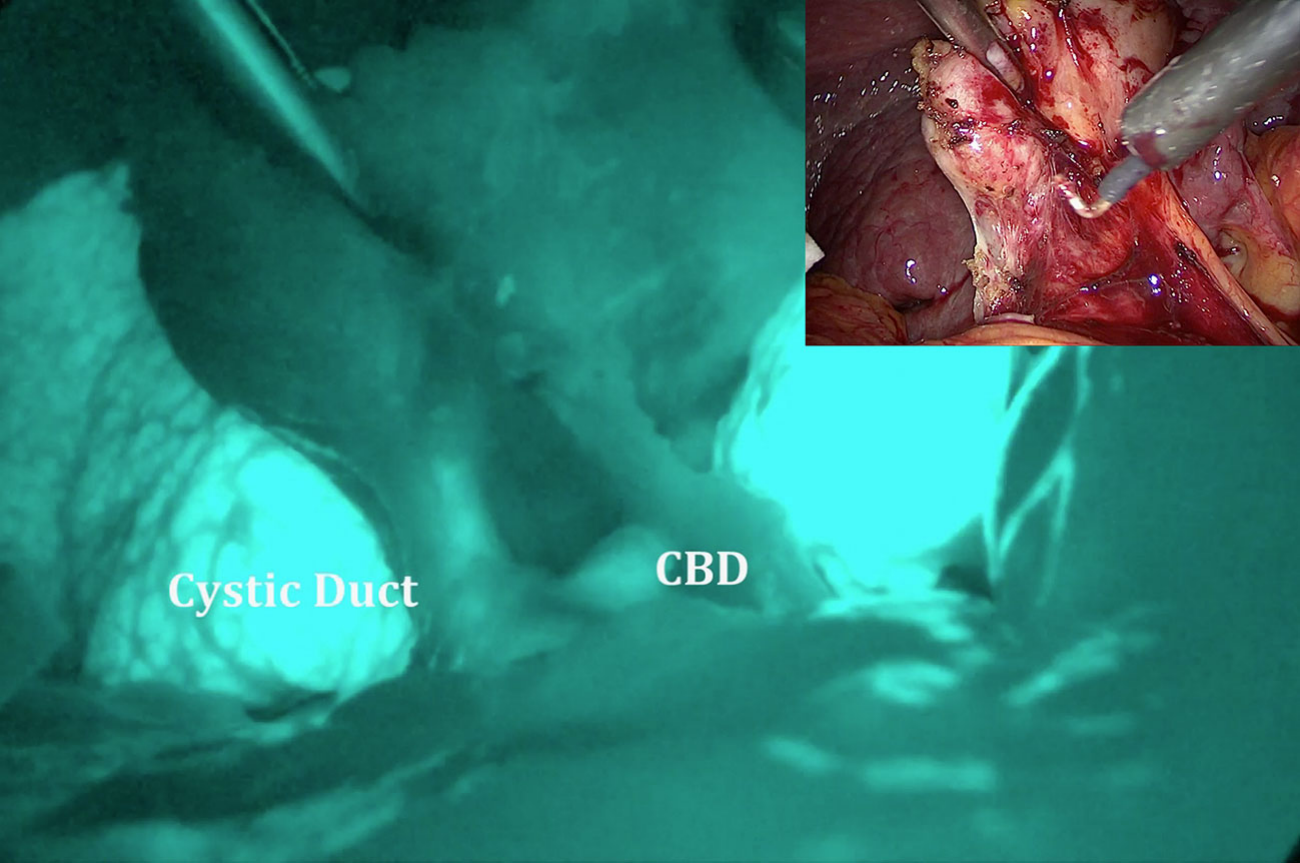
Use of ICG-enhanced fluorescence during laparoscopic cholecystectomy for acute cholecystitis. Insert in *upper right corner* shows the operative view using standard light

We were able to identify the biliary anatomy in all cases (100 % sensitivity), especially the cystic duct-common bile duct junction, irrespectively of whether the tissues were normal or inflamed (Figs. 1, 2).

**Fig. 3:**
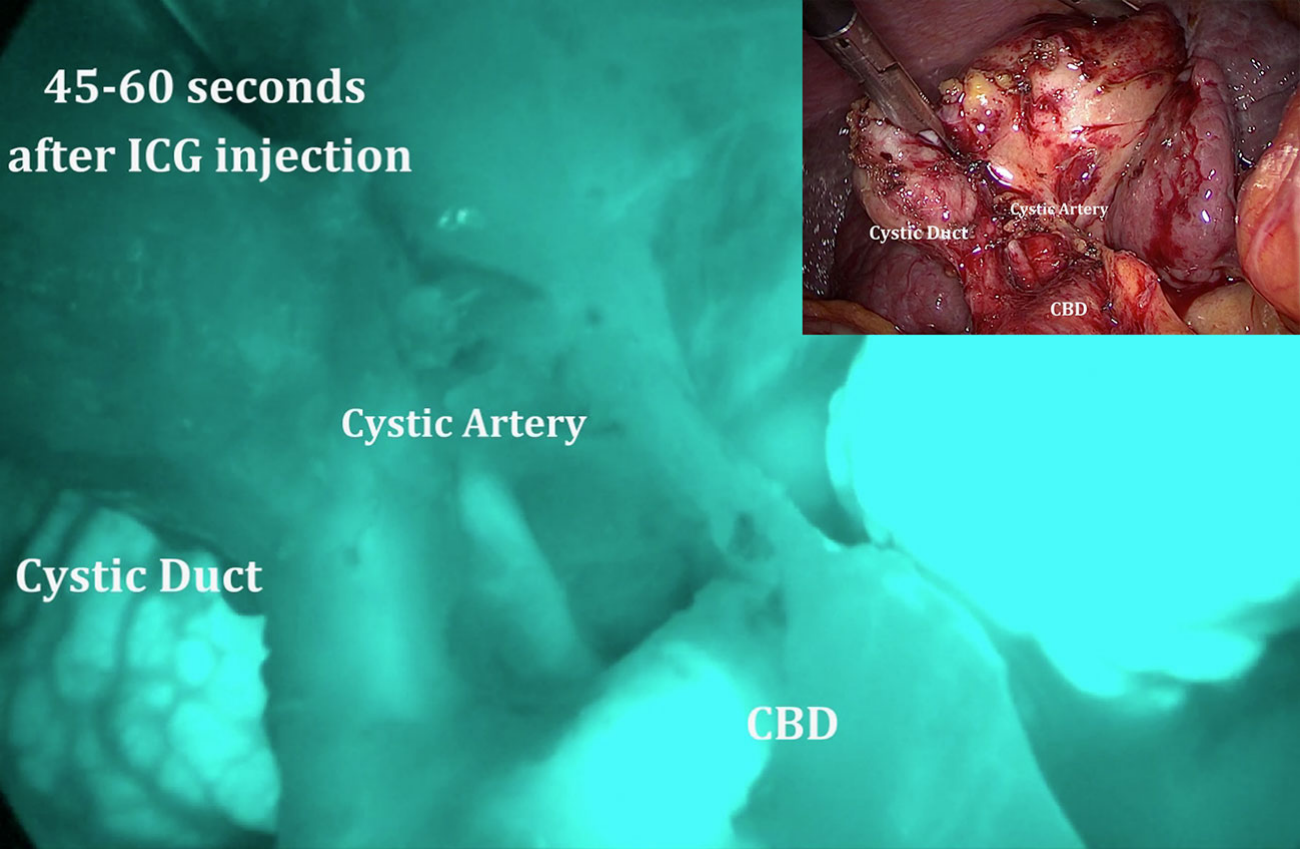
Identification of the cystic artery during laparoscopic cholecystectomy for acute cholecystitis. In the *upper right corner*, the operative view using standard light

If the vascular anatomy of the cystic artery required clarification, a small bolus of 2–3 ml of 0.4 mg/ml/kg was injected. Fluorescence appeared at the level of the Calot’s triangle defining the cystic artery (Fig. 3) after 60 s (mean delivery time 63 ± 12 s) and lasting for a mean time of 32 ± 9 s.

The mean operative time was 54 ± 13 min.

There were no adverse reactions to the ICG injection, and we reported no intra-operative or post-operative complications.

### Fluorescence-guided colorectal resection

ICG-enhanced fluorescence was used during laparoscopic colorectal resection in order to verify the adequate perfusion of the large bowel prior to anastomosis.

Once injected into a peripheral or central vein, ICG became fluorescent under NIR light, providing a “real-time” confirmation of the bowel perfusion. Thus, this helps to define the point of resection after mesenteric division as well as demonstrates the presence of an ischemic or “non-optimal” perfusion before performing the anastomosis.

We performed 38 ICG-guided colorectal resections including 15 left sigmoid resections (12 for cancer and 3 for diverticular disease), 12 anterior resections with total mesorectal excision, 8 right, and three transverse colectomies for cancer, in 34 patients (21 male, 13 female, mean age 63 ± 12 years).

In all cancer cases, a medial to lateral approach with high vessel ligation was used [24]. In 11/13 female patients, the specimen was extracted though a colpotomy, while in the remaining two cases, a supra-pubic mini-laparotomy was used.

**Fig. 4:**
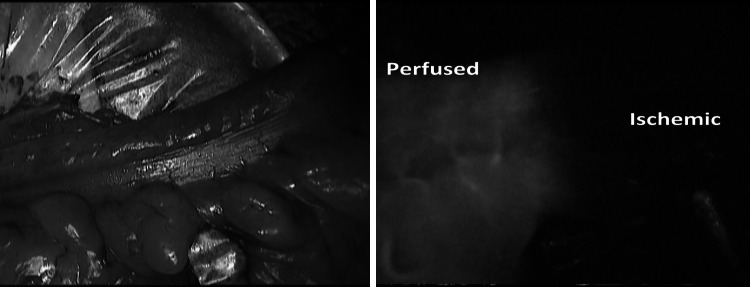
Identification of the ischemic bowel after mesenteric division during laparoscopic anterior resection. On the *left* *side*, the external view using standard light (notice no difference in the two segments). On the *right* *side*, the view using near infra-red light after injection of 5 ml of ICG

In order to study the perfusion of the bowel, ICG injection was performed using 2 bolus of 5 ml each at a concentration of 0.4 mg/ml/kg: the first after the division of the mesentery to help choose the best perfused site for resection and the second just before performing the anastomosis to ensure adequate vascularization (Fig. 4).

In case bowel, division was performed extra-corporally; as in most of the left-sided resections, in order to identify the fluorescence, the operative room should be completely darkened in order to identify the fluorescence since external light impairs fluorescence detection by the camera.

**Fig. 5:**
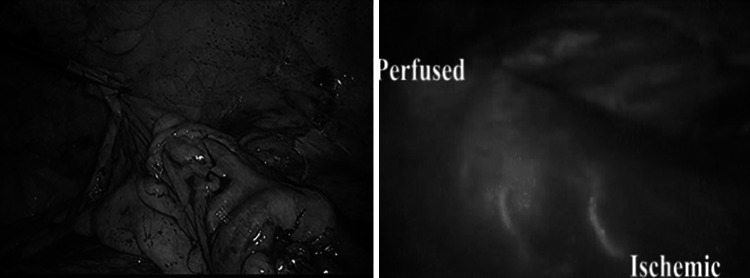
Fluorescent perfusion control during anterior resection with identification of a distal ischemic part using near infra-red light after injection of 5 ml of ICG (*right* *side*)

In all 38 cases, we were able to obtain a real-time image that demonstrated the perfusion of the bowel. In one case of anterior resection with trans-vaginal specimen extraction, when ICG was injected only after placing the anvil of the circular stapler, an ischemic area of the distal bowel was revealed, requiring re-resection and re-positioning of the anvil (Fig. 5) that was successfully performed laparoscopically.

We reported no intra-operative or injection-related complications, and we observed no anastomotic leaks.

**Fig. 6:**
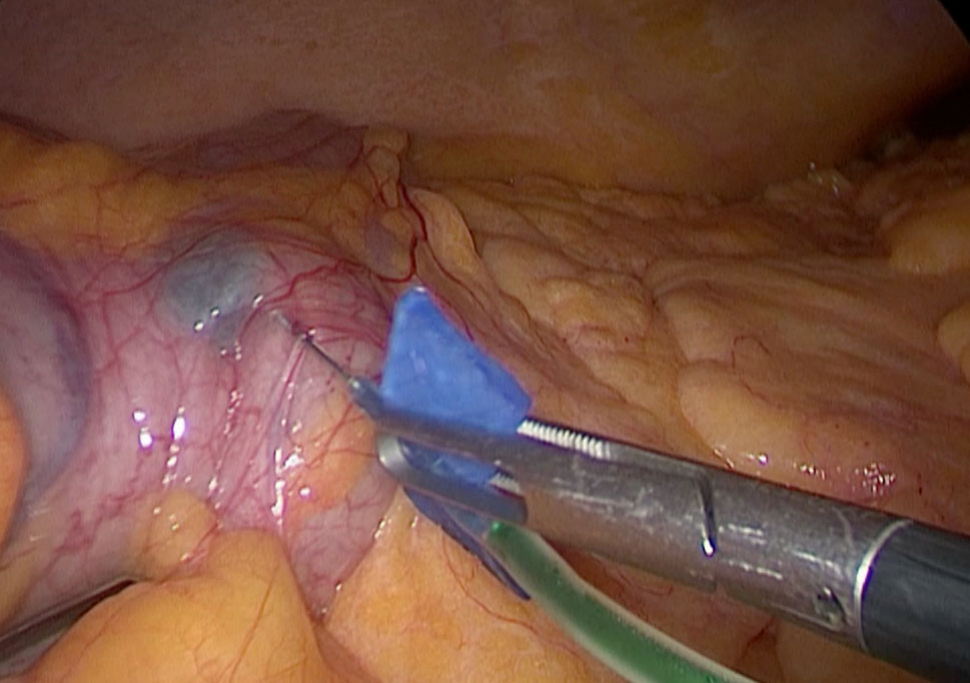
Peri-tumoral lymphatic mapping after ICG injection during laparoscopic *right* colectomy

**Fig. 7:**
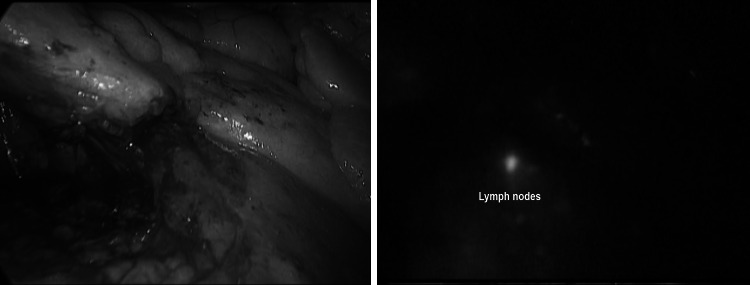
ICG-enhanced fluorescent lymphatic mapping during laparoscopic *right* colectomy. On the *left* *side*, the view using standard light. On the *right* *side*, identification of lymph node at the origin of the ileo-colic vessels using NIR light

In order to study the lymphatic drainage of the colon, peritumoral injection of 20 % albumin-diluted ICG was performed during right colectomy (Fig. 6) in four patients and identification of the lymphatic pathway as well as one residual node at the origin of the ileo-colic vessels was visualized (Fig. 7)

### Fluorescence-guided lymphadenectomy

We performed ICG-guided inguino-iliac/obturator lymph node dissection for metastases originating from left lower limb melanoma previously removed by plastic surgeons in three patients.

In these cases, indocyanine green was diluted with 20 % albumin and injected at a concentration of 0.5 mg/ml/kg around the scar of the primary lesion 15–20 min before surgery.

The patients were placed in lithotomy position, and four trocars were inserted, as in standard left colectomy [25]. The sigmoid colon was mobilized, and the left iliac vessels were exposed. The first part of the procedure was carried out using only standard light with complete removal of the fatty-lymphatic tissue around the iliac vessels and obturator nerve.

**Fig. 8:**
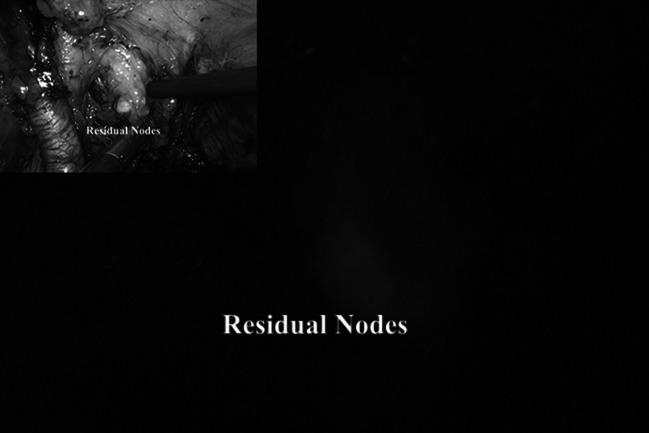
The use of ICG during inguino-iliac/obturator lymph node dissection. On the *left* *side*, the view using standard light on the *right* the lymph node enhancement with ICG

At this point, after switching to NIR light, the residual nodes could be easily identified by fluorescence (Fig. 8) and removed. Obviously, the inguinal lymph nodes dissection was performed via an “open” technique.

The mean operative time was 135 ± 22 min. There were no intra- or post-operative complications. The mean number or removed lymph nodes were 39 ± 12.

### ICG-enhanced fluorescence to study vascular anatomy and parenchymal perfusion

ICG can be used both to clarify the vascular anatomy as well as to identify ischemic parenchyma in various clinical situations.

**Fig. 9:**
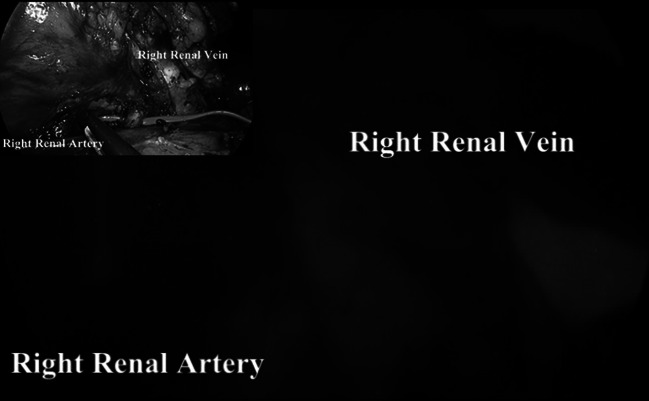
Vascular anatomy study using ICG-mediated fluorescence during *right* laparoscopic living-donor nephrectomy after injection of 5 ml of ICG. In the *upper left corner*, operative view using standard light

**Fig. 10:**
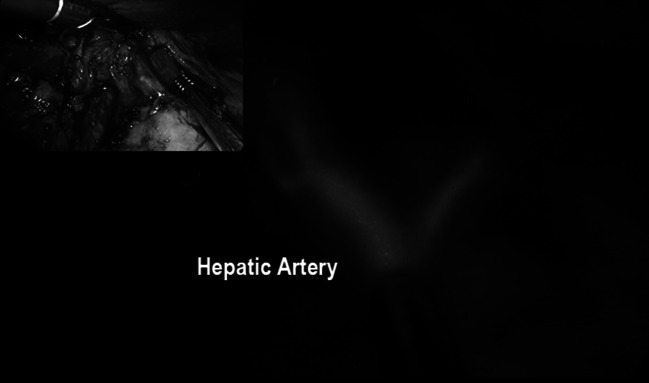
Vascular anatomy study of the hepatic artery using ICG-mediated fluorescence during laparoscopic liver resection using near infra-red light after injection of 5 milliliters of ICG. In the *upper left corner*, the view using standard light

**Fig. 11:**
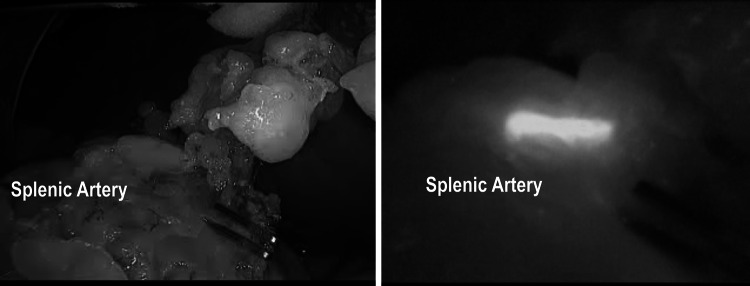
Vascular anatomy study of the spleen using ICG-enhanced fluorescence during laparoscopic splenectomy. On the *right side*, view using near infra-red light after injection of 5 ml of ICG. On the *left side*, view using standard light

**Fig. 12:**
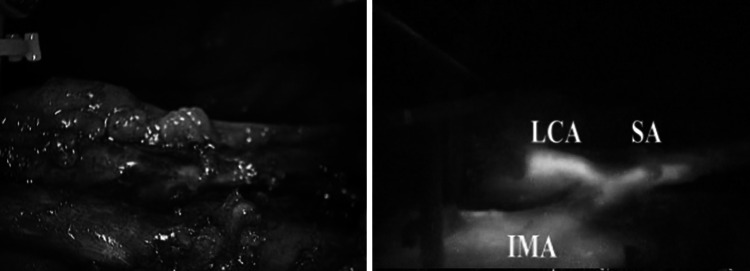
Vascular anatomy study of the inferior mesenteric artery using ICG-mediated fluorescence laparoscopic treatment of type II endoleak. On the *left side*, view using standard light. On the *right side*, view using near infra-red light after injection of 5 ml of ICG

We used ICG-enhanced fluorescence to clarify the vascular anatomy during laparoscopic living-donor nephrectomy (8 cases) and laparoscopic kidney autotransplantation for renal artery aneurysm (1 case) (Fig. 9), liver resection (2 cases) (Fig. 10), splenectomy (2 cases) (Fig. 11), and laparoscopic ligation of the inferior mesenteric artery of type II endo-leak after endovascular repair of aortic aneurysm (2 cases) (Fig. 12).

For these procedures, ICG was injected in small boluses of 3–5 ml each (0.4 mg/ml/kg), and the real-time fluorescence was recorded.

**Fig. 13:**
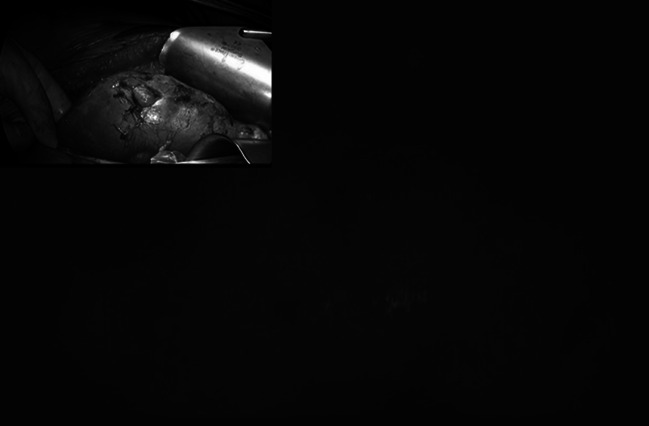
Parenchymal perfusion assessment of the kidney after transplantation (injection of 5 ml of ICG). In the *upper left corner*, the operative view using standard light

In case of kidney transplantation, fluorescence distribution inside the parenchyma was also used to confirm an adequate perfusion of the organs after vascular anastomosis (Fig. 13).

## Discussion

Since ICG is excreted virtually unchanged by the bile, the most obvious application is the visualization of the biliary tree. Indeed, iatrogenic bile ducts injury is still one of the most dangerous complications of cholecystectomy, with an incidence between 0.4 and 0.7 %, and recently reported to be as high as 1.3 % [26], generally due to misinterpretation of biliary tract anatomy [27–29]. Careful and meticulous dissection of the Calot’s triangle, achieving the so-called “critical view of safety” and maybe performing intra-operative cholangiogram, possibly combined [29] have been demonstrated to be able to keep bile ducts lesion as low as possible [27, 28].

Nevertheless, all the above maneuvers, including intra-operative cholangiogram, require a certain degree of dissection in potentially dangerous areas when the anatomy is not straightforward. The would-be accidental bile duct injuries cannot be prevented but only demonstrated by intraoperative cholangiogram.

As shown by our experience (Figs. 1, 2, 3), using ICG-enhanced fluorescence, we were able to perform a sort of “virtual” cholangiography at the very start of the procedure, allowing the surgeon to identify either the normal anatomy or possible anatomic variations in normal settings or in potentially dangerous situations (i.e., the presence of inflammatory tissue), areas to be respected until the dissection allows a better identification of the different structures (Figs. 2, 3). Ishizawa et al. [28] reported cystic duct and common hepatic duct visualization of 100 and 96 % previous dissection and 100 % of both after dissection. These results are also reported by other authors using both NIR and laser beam systems during standard laparoscopic or robotic multiport and single port cholecystectomy [30–33]. Of note, in our study, as in others [28], the sensitivity of ICG in the recognition of the cystic and common bile ducts (or their junction) was 100 %

As concerns the exact dose and concentration of ICG to be given to patients, most authors use 0.2–0.5 kg body weight [13, 34]. Morita et al. [35] used 2.5 mg but did not state the dilution or the volume. In our experience, 5 ml of 0.3–0.4 mg/ml/kg provided adequate concentration in the bile hence an adequate visualization of the biliary tree.

The time between ICG injection and presence of the dye in the bile has also been the topic of several publications [21, 22]. More than 95 % of ICG is captured by hepatocytes and excreted into bile within 15 min of injection [22]. Fluorescence of the liver and bile ducts can last up to 6 h after intravenous injection of ICG [21, 28]. The interval is related to liver function: organs with poor function and cirrhosis [36] will take much longer to extract ICG from the blood to the bile, but on average, we can conclude that 10–15 min is usually sufficient.

As demonstrated in one of our cases, an extra bolus of ICG can be used to clarify the vascular anatomy at the level of the Calot’s triangle; although mentioned as being possible by Alander et al. [13], to the best of our knowledge, this particular clinical application has not been reported in the literature yet. These authors recommend waiting 15 min before injecting the second bolus [13].

A further interesting clinical application of fluorescence is the possibility to study in real-time perfusion of organs and bowel prior to or after anastomosis.

Among the risk factors for anastomotic leakage, one of the most important and well-recognized most dreadful complications is poor local tissue oxygenation secondary to inadequate anastomotic vascular perfusion [37, 38].

Presently, either subjective clinical findings such as tissue coloration, pulsation of marginal vessels, temperature, bleeding from marginal arteries, peristalsis, or objective or Doppler measurements [39] can be used to confirm the adequate perfusion of bowel.

As demonstrated with our experience, a simple injection of few milliliters of ICG allows to have a real-time evidence of adequate perfusion of the bowel prior to proximal transection, after division of the mesentery and before the completion of the anastomosis (Fig. 4).

By comparison, more than 10 min is required in order to obtain an ischemic demarcation of the bowel visible to standard light after vessel division, while ischemia of the colon is immediately evident using fluorescence. In one of our cases, ICG-mediated fluorescence allowed to identify an unexpected ischemic distal segment requiring re-resection and preventing a highly compromised intestinal segment, probably at high risk for post-operative leakage (Fig. 5).

Few studies on the use ICG fluorescence imaging to assess the vascularization of colorectal anastomosis have been published to date.

In a retrospective study, Kudszus et al. [40] used laser fluorescence angiography with ICG to visualize colorectal anastomoses and were able to demonstrate a 60 % reduction rate in anastomosis revision, similar to the experience reported by Jafari et al. [41]. While these are small-size studies and case series, the results are very promising. The recently completed multicenter study in the US [42] has also shown very encouraging results.

The assessment of organ perfusion and ischemia using fluorescence has also potential applications for other organs such as the kidney after transplantation, liver during resection [43], spleen for partial splenectomy, and gastric conduit during esophagectomy [42], to mention a few.

As for other compounds, ICG can also be used as a dye for mapping the lymphatic drainage from different organs [13].

ICG-mediated fluorescence has been proposed for sentinel lymph node biopsy in breast surgery and for melanoma using a specifically designated camera for “open” surgery [44, 45]. In these cases, some authors recommend diluting ICG with 20 % albumin in order to guarantee a correct diffusion into the lymphatic vessels. However, a recent randomized controlled study in breast cancer was unable to detect any statistically significant difference in efficacy [46].

In laparoscopic surgery, possible clinical applications include identification of intra-abdominal sentinel lymph node for melanoma or to help during lymphadenectomy in case of metastatic melanoma [18], prostate [47], or endometrial cancer [48].

In colorectal surgery the peri-tumoral injection of ICG can be used to study lymphatic mapping that might be interesting in case of right sided tumors, known to have highly variable lymphatic drainage [49] or for sentinel lymph nodes biopsy in early stage rectal cancers [50].

In our experience, fluorescence can be also applied to facilitate the vascular dissection in specific or unclear situations when anatomic variables can be expected such as in case of nephrectomies, liver resections, vascular surgery, and splenectomy (Figs. 9, 10, 11) and metastatic melanoma (Fig. 8). In such cases, the use of ICG allows to obtain a “real-time” pathway of the vessel distribution that can be of help during the dissection.

In the future, superposition of transparent light images with those obtained by fluorescence (augmented reality) might improve bile duct dissection even more.

## Conclusions

ICG-enhanced laparoscopic surgery can be applied during different procedures offering to the surgeon additional information on anatomy, perfusion, or lymphatic drainage.

Our experience demonstrated the potential benefits and safety of this new technology.
